# Age-Dependent Differences in the Antiviral Response of the Respiratory Epithelium

**DOI:** 10.3390/v18070780

**Published:** 2026-07-16

**Authors:** Leah Setar, Bria Coates

**Affiliations:** 1 Ann & Robert H. Lurie Children’s Hospital of Chicago, Chicago, IL 60611, USA; bcoates@luriechildrens.org; 2 Department of Pediatrics, Northwestern University, Chicago, IL 60611, USA

**Keywords:** respiratory viral infections, respiratory epithelium, innate immune response, interferon signaling, pediatric immunity

## Abstract

Age is an established risk factor for severe viral respiratory infections, yet the mechanisms driving increased severity of illness in infants and older adults remain incompletely understood. The respiratory epithelium is the primary target of viral infection and how it orchestrates the immune response may be a key determinant of clinical outcomes. The profound age-related difference in susceptibility to severe Coronavirus disease 2019 (COVID-19) brought increased attention to the epithelial response to viral infection in children and adults, adding significant data to the preexisting body of work largely focused on influenza and respiratory syncytial virus (RSV). This review synthesizes current knowledge on the structural and functional differences in the respiratory epithelium between children and adults at baseline and during viral infection. We review the variable and heterogenous human studies in addition to in vitro and animal model data to identify key gaps in current knowledge. Here, we advocate for a more complete understanding of age-dependent differences in the respiratory epithelial response to viral infection to uncover therapeutic targets for prevention and treatment of viral-associated respiratory failure.

## 1. Introduction

Viral respiratory infection remains a leading cause of hospitalization, especially at the extremes of age [1]. Though age is a risk factor for severe viral respiratory infections, age-dependent susceptibility to severe disease is not consistent amongst different viruses, and a wide spectrum of severity is seen in all ages. In both influenza and respiratory syncytial virus (RSV) infection, young children and elderly adults have increased risk of severe disease [2,3]. In contrast, Severe Acute Respiratory Syndrome Coronavirus 2 (SARS-CoV-2) infection, responsible for the Coronavirus disease 2019 (COVID-19) pandemic, primarily causes severe disease in adults, not children [4,5]. Although immunizations, prophylactic antibodies, and antiviral medications are now available for some viruses, infection remains common and treatment for viral-associated respiratory failure is mostly limited to supportive care. Two individuals with the same virus and risk factors can have drastically different clinical presentations, suggesting differences in the host response drive clinical outcomes. Recent data have identified many differences in the antiviral response between adults and children, but there remains much to learn about how these differences are helpful or harmful during viral infection.

The respiratory epithelium, spanning from the nose to alveoli, is the primary site of viral respiratory infection and the host immune response [6]. A coordinated, balanced immune response to viral infection is crucial. If this response is muted, excessive, or delayed, it can lead to lung damage and severe clinical outcomes [6]. If efficient and timely, the local response in the nasal epithelium can coordinate viral clearance and prevent spread to the lower respiratory tract [7]. Many studies that focus on age-related differences in the antiviral response assess peripheral blood, which does not represent the response of the epithelium [8]. Here, we review baseline differences in the respiratory epithelium between children and adults, as well as differences in the response to viral infections. Improved understanding of these age-dependent differences in the respiratory epithelial compartment before and during viral infection may help us understand the distinct mechanisms of severe disease in each age group.

## 2. Baseline Differences Between Adult and Pediatric Respiratory Epithelium

In addition to the lungs and airways of children being smaller than those of adults, there are differences in development and cell composition that are important context for understanding differences during viral infections. Respiratory epithelial cells are the first line of defense against inhaled pathogens. The conducting airways, including the nasal and bronchial regions, are lined by a ciliated epithelium mainly composed of ciliated cells, mucous-secreting goblet cells, and basal cells, which function as the stem cells of the epithelium [9,10]. Ciliated cells, necessary for mucociliary clearance to remove pathogens and particles from the airway, are the primary target of respiratory viruses [11,12,13]. Ciliary beat frequency and the efficiency of mucociliary clearance is decreased in aging adults, though differences within pediatric age groups remain unclear [14,15,16]. At the distal end of the respiratory tract lies the alveolar epithelium, covering a large surface area for gas exchange lined by alveolar type 1 and 2 cells, both of which can be infected by most respiratory viruses [17,18]. Alveolar development begins before birth and continues into early adulthood [19,20,21]. This is important in relation to lung injury, as younger lungs may be more capable of repair and formation of new alveoli, but inflammation or injury during alveolarization can interfere with normal lung development [19]. In addition to the epithelial cells in the respiratory tract, there are resident immune cells throughout as well as recruited immune cells when a pathogen is present [6]. The interactions between these recruited immune cells and respiratory epithelial cells are crucial for a coordinated antiviral response.

The major cell types of the respiratory epithelium are similar between adults and children, with minor differences depending on the region. In the nasal mucosa, there is a decreased proportion of goblet cells and increased proportion of ciliated cells with age, while the relative percentage of basal cells tends to be higher in adults [22,23,24]. However, when infant and adult nasal epithelial cells were grown at air–liquid interface (ALI), infant cells grew thicker with prominent basal cells, while adult cells had prominent goblet cells [25]. In contrast, tracheobronchial biopsies obtained from adult and pediatric donors did not show a significant difference in the proportion of basal, secretory or ciliated cells [26]. Further altering these composition differences, the NOTCH signaling pathway, the molecular pathway involved in determining ciliated or goblet cell fate, can be triggered excessively in viral infection [27,28]. Studies directly comparing the composition of adult and pediatric alveolar epithelium are limited, as these samples are quite difficult to access.

The way in which pediatric respiratory epithelial cells grow in culture also differs from adult cells. Tracheal basal cells from children proliferate more readily than tracheal basal cells from adults in culture [26]. Further, pediatric basal cells outcompete adult cells in mixed cultures, supportive of their increased survival and proliferative capacity [26]. Similar findings are seen in nasal cells, with an increased number of Ki67^+^ proliferating cells seen in nasal cultures from young children than from adults [29,30]. In addition, a rare regenerative epithelial cell that emerged in adult patients with COVID-19 was subsequently identified in the epithelium of healthy children, suggesting a higher cell turnover state in children at baseline [10]. Taken together, while composition of the respiratory epithelium is overall similar across ages, continued lung development, increased proliferative capacity and higher cell turnover is evident in pediatric epithelium. How this may affect susceptibility to severe viral infections is unknown.

The pediatric respiratory epithelium has higher baseline immune activity than the adult respiratory epithelium. There is a significantly greater proportion of immune cells at the epithelial barrier in healthy children as compared to healthy adults [22,31]. Single-cell analysis from nasal swabs reveals that most of these immune cells are Tissue-Resident memory T cells (Trms), though the decline in immune cells seen with age mostly affects monocytes [23]. Uninfected juvenile mice have an increased number of alveolar macrophages in the distal lung compared to adult mice, suggesting intrinsic age-associated differences affecting immune cell presence in the alveoli, even in the absence of pathogens [32]. In addition to increased presence of immune cells, healthy children display higher basal expression of viral RNA sensors, including MDA5 and RIG-1, in upper airway epithelial cells, macrophages, and dendritic cells compared to healthy adults [22,31]. When human nasal epithelial cells from healthy individuals are grown at ALI, MDA5 transcription is also higher in children than adults [33]. However, when bronchial epithelial cells are grown at ALI, there is no significant age-associated difference in MDA5 and RIG-I, suggesting these differences are not consistent throughout various locations in the respiratory tract [34]. This data overall suggests both cell-intrinsic and cell-extrinsic factors may drive age-related differences in baseline immune cell presence and reactivity in the respiratory epithelium.

Interferons (IFNs) play a key role in the early antiviral response but can also perpetuate inflammation and tissue damage [35]. Type I interferons (IFN-α and IFN-β) are secreted by both epithelial and immune cells while type III interferon (IFN-λ) is mostly restricted to the epithelium [35]. Type II interferon (IFN-γ) is produced by innate-like and adaptive immune cells, classically CD4^+^ T cells [35]. A growing body of literature has focused on interferons across the lifespan, though the emphasis has been on aging populations and the systemic response [36]. Studies assessing baseline interferon activity of the epithelium are limited, but generally suggest that interferon activity is higher in the respiratory epithelium of children and decreases with age. Transcriptomic analysis from upper and lower airways found that type I and III interferon gene expression and downstream signaling were higher in healthy children than in healthy adults [37,38]. In contrast, a separate study of nasopharyngeal samples found mixed age-related differences in type I interferon levels, with decreased IFN-β expression in pediatric samples compared to adult and no significant difference in IFN-α [39]. Instead, nasal samples from uninfected children had higher levels of IFN-γ and proinflammatory cytokines, including interleukin-6 (IL-6), interleukin-10 (IL-10), and Tissue Necrosis Factor (TNF), than from adults [31]. When pediatric upper and lower airway transcriptomes were compared, baseline enrichment for interferon signaling was more pronounced in lower bronchial tissue than nasal tissue, suggesting potential tissue-specific age-dependent differences [38].

**Figure 1:**
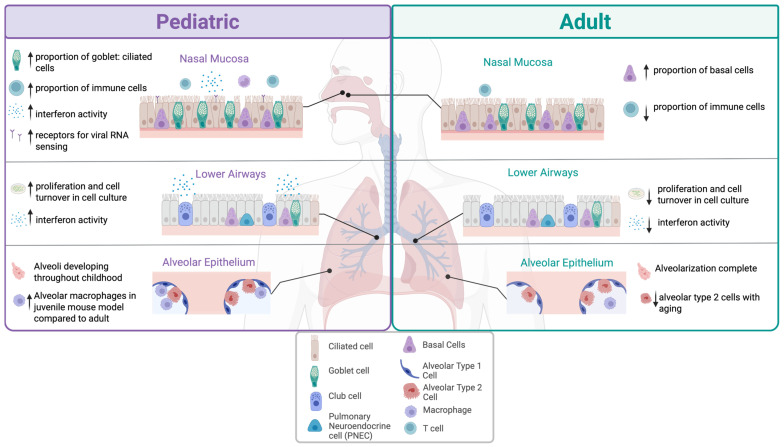
Summary of studies comparing pediatric and adult respiratory epithelium at baseline. The lower airways have similar proportions of ciliated, goblet and basal cells. Studies comparing alveolar epithelium between children and adults are limited. Created in BioRender. Setar, L. (2026) https://BioRender.com/bu44pp0.

It has been hypothesized that higher baseline immune activity in the airways of healthy children is driven by the frequency with which children are infected with respiratory viruses. Over half of children tested at a routine doctor’s office visit had a respiratory virus on nasal testing [40], and children are much more likely to have coinfection with multiple viruses than adults when ill [41]. While undiagnosed or recent viral infections may contribute to some of the observed differences in baseline immune activation, the persistence of age-associated differences in pathogen-naive animal models suggests that there are also intrinsic differences [29,30,32]. These studies directly comparing the uninfected adult and pediatric respiratory epithelium are summarized in Figure 1.

## 3. Differences in Viral Infectivity of Adult and Pediatric Respiratory Epithelium

Multiple studies failed to find a significant difference in viral load or viral clearance in nasal samples from adults and children with SARS-CoV-2 infection [42,43,44,45]. In cultured nasal epithelial cells, one study found that SARS-CoV-2 viral load was higher in adult cultures than pediatric cultures at 48 h post-infection, while another found no significant difference over the first 5 days of infection [24,46]. In cultured human bronchial epithelial cells, there was no difference in viral replication of SARS-CoV-2 between adult and pediatric cultures, though one study identified that a small number of cultures were resistant to viral replication, which was more common in pediatric-derived cultures [34,47]. Another study demonstrated that the ancestral and delta strains of SARS-CoV-2 replicated to much lower levels in pediatric nasal epithelial cells compared to adult nasal epithelial cells, with a less pronounced difference in the more recent Omicron strain [30]. This suggests that the SARS-CoV-2 virus evolved to evade protective responses in the pediatric epithelium that restrict its growth.

In contrast to SARS-CoV-2, viruses associated with increased severity of illness in children tend to replicate faster or to higher levels in cultures of pediatric cells compared to adult cells. In tracheobronchial epithelial cultures from rhesus monkeys, infant epithelium infected with influenza had higher viral titers than adult epithelium infected with the same dose [48]. However, in children and adults infected with influenza, nasal viral titers and the timing of peak viral load were similar throughout infection [8,49]. In human nasal organoids, RSV viral load peaked on day 2 post-infection in children and on day 5 in adults, but with similar magnitudes [50]. In bronchial epithelial cells from adults and children infected with human metapneumovirus (hMPV), viral titers were also higher in pediatric cells during the first 3 days of infection [51]. In human nasal epithelial cultures infected with rhinovirus, associated with childhood wheezing, cell cultures from children showed higher viral loads than adults when infected, which was more pronounced at higher doses [33]. Additional studies are needed to determine if age-associated differences in magnitude and/or timing of viral replication are a driver or a consequence of age-dependent immune responses in the respiratory epithelium.

Viral binding was a popular point of research early in the COVID-19 pandemic. Multiple studies sought to identify age-related differences in the major SARS-CoV-2 entry proteins, Transmembrane protease, serine 2 (TMPRSS2) and Angiotensin-converting enzyme 2 (ACE2), to explain the stark differences in disease severity depending on age. Data remains conflicting, with some studies showing that ACE2 and TMPRSS2 increase with age [38,52,53] and several others finding no difference between adults and children [10,22,34,46,54,55,56]. Neuropilin-1 (NRP1), a coreceptor that potentiates infection, was lower in nasal swabs from children but not different between in vitro nasal cultures from adults and children [56]. Studies are sparse in other viruses. Higher expression of the α2-3A sialic acid influenza receptor was found in children than adults [57]. Similar RSV binding to neonatal and adult tracheal aspirate cells was reported in culture [58]. Based on this limited data, age-associated differences in viral binding to epithelial cells does not appear to fully explain differences in susceptibility to viral infection.

In vitro experiments have also revealed age-related differences in viral tropism and the susceptibility of epithelial cells to the cytopathic effects of RSV infection. In adult human nasal organoids, RSV predominantly infects ciliated cells, but in pediatric nasal organoids, it has broader cell tropism, infecting aberrant basaloid cells and rare cell types like ionocytes and tuft cells [29]. Neonatal tracheal-derived epithelium infected with RSV showed increased cytopathy, mucous hyperplasia, viral spread, inflammatory signaling, and apoptosis when compared to adult-derived epithelium [58]. These studies support an intrinsic susceptibility of pediatric epithelial cells to RSV-induced damage and cell death. RSV also suppresses proliferation of adult human nasal organoids more than pediatric human nasal organoids [29]. Additional studies are needed to investigate whether increased cell death and cell proliferation in children with RSV infection may lead to increased cell turnover and sloughing of cells into the airways, contributing to the pathology of clinical bronchiolitis and respiratory failure.

## 4. Innate Response of the Epithelium and Age

The epithelial innate immune response differs with age, virus, and tissue. Human samples obtained from the nose during SARS-CoV-2 infection have not consistently shown age-related differences in the interferon response [10,22,37,39,54,55,59,60]. However, clinical studies interrogating the interferon response are limited by unknown time from infection to sample collection, low sample numbers, and potential for concurrent or recent viral infection, especially in children. The human studies directly comparing the nasal antiviral response between adults and children are summarized in Table 1.

In vitro studies suggest potential tissue-specific, age-associated differences in the epithelial immune response to SARS-CoV-2. Nasal cells cultured at ALI from children infected with SARS-CoV-2 showed increased interferon-stimulated gene (ISG) expression and antiviral signaling compared to adult cells, as well as a higher number of Goblet 2 inflammatory cells, associated with type I IFN signaling [24,30,46,61]. Conversely, adult bronchial epithelium cultured at ALI showed a stronger proinflammatory transcriptional response than pediatric bronchial epithelium [47]. Studies of alveolar epithelial cells are limited and cell lines derived from lower airway cells are overwhelmingly from adult donors. Overall, these studies suggest that a more robust epithelial response to SARS-CoV-2 in the nasal cells of children may control viral replication and prevent extension to the lower airways, while the greater proinflammatory response of adult epithelial cells in the lower airways may increase tissue damage and drive the inflammatory pneumonia characteristic of COVID-19. Further studies are needed to better understand how lower airway and alveolar cells respond to SARS-CoV-2 in children.

The epithelial response to respiratory viruses which significantly affect the pediatric population varies with age and tissue type. In nasal organoids infected with RSV, inflammatory cytokines were higher in pediatric-derived cells [50], though this was not reproduced in another study grown at ALI [25]. Nasal lavage fluid from children and adults with influenza demonstrated that inflammatory cytokines inversely correlated with age, even when adjusted for viral load [8]. Juvenile mice with influenza infection had twice the levels of IFN-β in bronchoalveolar lavage fluid compared to adult mice with similar viral titers [32]. In contrast, in cultured bronchial epithelial cells infected with RSV or hMPV, levels of IFN were similar early, but adult cells had higher Type I IFN, Type III IFN, and ISG expression late in infection [51]. Further, in airway epithelial cell cultures from rhesus monkeys infected with influenza, IFN-α response and Class II Major Histocompatibility Complex Transactivator (CIITA), associated with immune activation, were lower in infant cultures early in infection compared to adult cultures [48]. In a mouse model infected with rhinovirus, neonatal mice had increased type 2 cytokine expression (IL-13, IL-4, and IL-5) as well as mucous-related gene expression compared to adult mice, with blunted expression of type II IFN response [62]. Nasal epithelial cells stimulated with Poly I:C, a viral mimic and Toll Like Receptor 3 (TLR3) agonist, had a more robust IFN response in cells from children than in those from adults, but with significant variability [38]. Collectively, these studies demonstrate that epithelial antiviral and inflammatory responses vary by age, anatomic location, and viral pathogen. Pediatric epithelial cells often exhibit stronger interferon-mediated antiviral responses than adult cells, which may help limit viral spread in some contexts, but may perpetuate inflammation in others. Findings across studies are not entirely consistent, underscoring the complexity of age-dependent epithelial immunity and the need for further investigation, particularly in pediatric lower airway and alveolar epithelial cells. Table 2 provides a summary of age-associated differences in cytokine expression during infection of experimental models.

Resident immune cells of the epithelial space as well as those recruited during infection play an important role in the antiviral response but can drive excessive inflammation and tissue damage. Though changes in circulating immune cells at the extremes of age are out of the scope of this review, a subset of studies have focused on the immune cells in the epithelial compartment during infection. In SARS-CoV-2 infection, single-cell sequencing of upper airway samples demonstrated that the proportion of immune cells in children, which was significantly higher than adults at baseline, remained relatively stable in infection, while adults experienced a large influx of immune cells into the nasal compartment [22]. Children were found to have more activated neutrophils and inflammatory monocytes in the nose during SARS-CoV-2 infection, as well as a distinct memory T cell population not found in adults [10,22]. Transcriptional analyses of nasal samples in SARS-CoV-2 infection showed inconsistent differences in epithelial–immune crosstalk between adults and children. One study found enrichment of genes associated with B cell recruitment and T cell activation in children compared to adults [59], while another found enrichment of genes involved in neutrophil activation and T cell signaling in adults compared to children despite similar viral reads and severity [54]. Children with mild SARS-CoV-2 infection also had an increased reduction in peripheral myeloid cells compared to adults, suggesting these cells may be migrating to the lungs [63].

In nasal fluid from children and adults with influenza, there was an age-specific skewing in monocyte subtype, with the relative frequency of CD14^+^CD16^−^ conventional monocytes negatively correlated with age and CD14^low^CD16^+^ “patrolling” monocytes positively correlated with age [8]. In juvenile mice with influenza infection, there were twice as many recruited Ly6c^+^CD64^+/−^ inflammatory monocytes in the lungs than in adult mice. This correlates with increased monocyte chemoattractant protein-1 (MCP-1), responsible for recruitment of monocytes and associated with severity of illness in pediatric influenza, that is not seen in adult mice [32]. Similarly, in bronchoalveolar fluid from mice with RSV infection, juvenile mice had a sustained increase in monocytes and neutrophils in the lung as well as MCP-1 and pro-inflammatory cytokines at 72 h post-infection, while the adult mice returned to baseline levels [64]. In mice infected with rhinovirus, neonatal mice recruited more Group 2 innate lymphoid cells (ILC2s) than adult mice which persisted after infection and are associated with mucous metaplasia and airway hyperresponsiveness [62]. Overall, children tend to have increased immune cells in the epithelium during viral infection, likely influenced by baseline and antiviral response differences in the epithelium related to age, potentially increasing severity of disease in some contexts.

## 5. Discussion

Though age has a clear association with clinical outcomes and the epithelial cell response in viral infections, the mechanisms by which age-dependent differences contribute to disease pathology remain incompletely understood. Increased proliferative capacity of the pediatric epithelium may play a role in the susceptibility of children to certain viral infections by providing more targets to sustain infection. Increased interferon activity and immune cell presence throughout the respiratory epithelium in children may lead to efficient viral clearance in SARS-CoV-2 infection, but may promote excessive inflammation and lung injury in other viral infections. There are significant gaps in understanding how the timing, magnitude, and location of viral replication and the host immune response differ with age, which may be key drivers of age-related differences in severity of viral respiratory infections.

Frequent viral exposures in children likely impact immune responsiveness throughout childhood. While immune memory was historically reserved for the adaptive immune system, it is now understood to be important in the innate epithelial response through functional, metabolic, and epigenetic changes that can last weeks to months [65,66]. Referred to as trained immunity, this non-specific, enhanced or repressed responsiveness of immune and epithelial cells following secondary exposure to a pathogen may be helpful or harmful [67,68]. Future studies focused on epigenetic and metabolic changes in the epithelium associated with age and clinical outcomes may shed light on how prior exposures affect disease severity. Heterogeneity of viral respiratory failure and small sample sizes in human studies, especially in pediatric cohorts, have made it difficult to identify consistent differences across studies. Further, access to lower airway samples in children is extremely limited and much is extrapolated from upper airway samples, which may respond differently to pathogens. Multicenter studies with larger sample sizes and studies utilizing model organisms will be necessary to better understand and confirm the differences seen in viral kinetics and the immune response between adults and children.

Though mortality remains low overall for viral respiratory infections, evidence shows that early life lower respiratory tract infections can have long-term effects on lung function, and additional exposures later in life can lead to further decline [69]. Additionally, hospitalizations for respiratory viral infections pose a huge cost to the healthcare system, lead to hospital-acquired comorbidities, and can cause immense stress for patients and families [70]. Understanding age-associated differences in the respiratory epithelial response and how exposures throughout life influence this response will uncover potential therapeutic targets to prevent severe lower respiratory tract disease and promote recovery from viral-induced respiratory failure.

## Figures and Tables

**Table 1:** Studies comparing human gene expression of the respiratory epithelium during viral respiratory infection in children compared to adults. IAV = influenza. mo = months. yr = years. NP = nasopharyngeal. IFN = interferon. ISG = interferon-stimulated genes. TNF = tumor necrosis factor. ED = emergency department.

Virus	Ages	Illness Severity	Method	Main Findings Comparing Adults to Children	Authors
SARS-CoV-2	Children ≤15 yr; adults 15–65 yr; elderly ≥65 yr	Asymptomatic, mild to moderate	qPCR of NP swabs	IFN-α transcript increased significantly in infected elderly, but not adults or children. IFN-β increased during infection in adults and elderly but not children. IFN-λ1 transcripts negatively correlated with age. ISG expression not significantly different with age.	Gilbert et al. 2021 [39]
SARS-CoV-2	Children ≤18 yr; adults ≥18 yr	Hospitalized, range of severity	Bulk RNA sequencing of NP swabs early in hospitalization	No significant difference found in the magnitude of IFN response with age within the first three days of hospitalization with SARS-CoV-2. In those with high viral load, children enriched for inflammatory cytokines compared to adults.	Koch et al. 2021 [54]
SARS-CoV-2	Children 1 mo–17 yr; adults 21–77 yr	Mild to moderate	Single-cell RNA sequencing of nasal samples	The magnitude of ISGs in epithelial cells was higher in infected children than adults in early and late infection.	Loske et al. 2022 [22]
SARS-CoV-2	Children ≤19 yr; adults ≥40 yr	Mostly non-hospitalized	Bulk RNA sequencing of NP swabs; previously published adult data set	Children showed upregulation of T and B cell activation; IFN-γ and inflammatory cytokine production genes compared to adults. No significant difference in Type I IFN response. Evidence of decreased ciliated cells and increased basal cells in children, suggestive of regeneration.	Mick et al. 2022 [59]
IAV	<6 mo; 6–23 mo; 24–59 mo; 5–8 yr; 9–12 yr; 13–17 yr; ≥18 yr	Asymptomatic and mild to severe	qPCR of nasal lavage fluid	Nasal lavage IFN-α, Monocyte Chemoattractant Protein-3 (MCP-3), and IL-6 were significantly higher in infants and young children than adults, even when adjusted for viral load.	Oshansky et al. 2014 [8]
SARS-CoV-2	Children ≤16 yr; young adults 17–40 yr; middle age 41–60 yr; older adults ≥60 yr	Asymptomatic and non-hospitalized children; adults mostly symptomatic or hospitalized	qPCR of NP swabs	IFN-α, β and λ did not differ among age groups. IFN-ε, ω negatively correlated with age. ISG15 and ISG56 expression increased with age.	Pierangeli et al. 2022 [37]
SARS-CoV-2	Children ≤18 yr; adults ≥18 yr	Symptomatic infection; more adults than children hospitalized	Bulk RNA sequencing of NP swabs on presentation to ED	Children enriched for ISG, inflammatory cytokine, and NLRP3 inflammasome gene expression compared to adults.	Pierce et al. 2021 [55]
SARS-CoV-2	Neonate ≤ 30 days; infant 30 days–1 yr; young child 2–6 yrs, child 6–12 yrs; adolescent 12–18 yrs; adult ≥ 18 yr	Asymptomatic to severe	Single-cell RNA sequencing of nasal, tracheal, and bronchial samples	Children had higher baseline epithelial IFN-α response which only slightly increased in infection, while adults had lower baseline levels and a larger increase during infection. Similar expression pattern for IFN-γ, TNF signaling, and neutrophil migration.	Yoshida et al. 2021 [10]
SARS-CoV-2	Children ≤ 18 yr; adults ≥ 18 yr	Asymptomatic to mild, sampled prior to symptoms	Bulk RNA sequencing of NP swabs	Children had enrichment of S100 family alarmin signaling compared to adults. IFN response similar. T cell activation downregulated in symptomatic children compared to asymptomatic; upregulated in symptomatic adults compared to asymptomatic.	Yue et al. 2025 [60]

**Table 2:** Cytokine differences with age in experimental models of viral infection. ↑ = increased in. IFN = interferon. ISGs = interferon-stimulated genes. NFkB = Nuclear factor kappa-light-chain-enhancer of activated B cells. IL-6 = interleukin 6. IL-8 = interleukin 8. IL-1β = interleukin 1 beta. TNF-α = tumor necrosis factor alpha. CXCL9 = C-X-C motif chemokine ligand 9. MCP-3 = monocyte chemoattractant protein 3. IL-13 = interleukin 13, IL-4 = interleukin 4, IL-5 = interleukin 5.

Cytokine(s)/Downstream Signaling	Pathogen	Model	Age Group Increased
ISGs, NFkB	SARS-CoV-2	Human nasal epithelial cells	↑ children [24,30,46,61]
Proinflammatory genes	SARS-CoV-2	Human bronchial epithelial cells	↑ adult [47]
IL-6, TNF-α, IL-8, IL-1β, IFN-λ, CXCL9, MCP-3	RSV	Human nasal epithelial cells	↑ children [50] Not reproduced [25]
IFN-α, IFN-β, IFN-λ, IL-6, IL-8, TNF-α	RSV hMPV	Human bronchial epithelial cells	Early—no difference ↑ adult in late infection [51]
IFN-α	Influenza	Rhesus monkey airway epithelial cells	↑ adult [48]
IFN-β	IAV	Mouse model	↑ juvenile [32]
ISGs	Poly I:C	Human nasal epithelial cells	↑ children [38]
IL-13, IL-4, IL-5	Rhinovirus	Mouse model	↑ juvenile [62]
IFN-γ, TNF-α	Rhinovirus	Mouse model	↑ adult [62]

## Data Availability

No new data were created or analyzed in this study. Data sharing is not applicable to this article.
